# Unusual Genomic and Biochemical Features of *Paenarthrobacter lasiusi* sp. nov—A Novel Bacterial Species Isolated from *Lasius niger* Anthill Soil

**DOI:** 10.3390/ijms26010067

**Published:** 2024-12-25

**Authors:** Alexandra A. Dymova, Maxim A. Kovalev, Artemiy S. Silantyev, Anna A. Borzykh, Pamila J. Osipova, Svetlana V. Poddubko, Vladimir A. Mitkevich, Dmitry S. Karpov, Natalia V. Kostina

**Affiliations:** 1 Institute of Biomedical Problems, Russian Academy of Sciences, 123007 Moscow, Russia; alexandra.dymova@mail.ru (A.A.D.); borzykh.anna@gmail.com (A.A.B.); osipova.pamila@yandex.ru (P.J.O.); poddubko@imbp.ru (S.V.P.); 2 Faculty of Soil Science, M.V. Lomonosov Moscow State University, Leninskie Gory, 1, 119991 Moscow, Russia; nvkostina@mail.ru; 3 Engelhardt Institute of Molecular Biology, Russian Academy of Sciences, 119991 Moscow, Russia; kovalev_maksim_2002@mail.ru (M.A.K.); mitkevich@gmail.com (V.A.M.); 4 The Institute of Personalized Cardiology, I.M. Sechenov First Moscow State Medical University (Sechenov University), 119991 Moscow, Russia; artsilan@gmail.com; 5 Center for Precision Genome Editing and Genetic Technologies for Biomedicine, Engelhardt Institute of Molecular Biology, Russian Academy of Sciences, 119991 Moscow, Russia

**Keywords:** *Lasius niger*, *Paenarthrobacter lasiusi*, genome analysis, heavy metal metabolism, lipidome, antibiotic resistance, nitrogen metabolism

## Abstract

The black garden ant (*Lasius niger*) is a widely distributed species across Europe, North America, and North Africa, playing a pivotal role in ecological processes within its diverse habitats. However, the microbiome associated with *L. niger* remains poorly investigated. In the present study, we isolated a novel species, *Paenarthrobacter lasiusi*, from the soil of the *L. niger* anthill. The genome of *P. lasiusi* S21 was sequenced, annotated, and searched for groups of genes of physiological, medical, and biotechnological importance. Subsequently, a series of microbiological, physiological, and biochemical experiments were conducted to characterize *P. lasiusi* S21 with respect to its sugar metabolism, antibiotic resistance profile, lipidome, and capacity for atmospheric nitrogen fixation, among others. A notable feature of the *P. lasiusi* S21 genome is the presence of two prophages, which may have horizontally transferred host genes involved in stress responses. *P. lasiusi* S21 synthesizes a number of lipids, including mono- and digalactosyldiacylglycerol, as well as steroid compounds that are typically found in eukaryotic organisms rather than prokaryotes. *P. lasiusi* S21 exhibits resistance to penicillins, lincosamides, fusidins, and oxazolidinones, despite the absence of specific genes conferring resistance to these antibiotics. Genomic data and physiological tests indicate that *P. lasiusi* S21 is nonpathogenic to humans. The genome of *P. lasiusi* S21 contains multiple operons involved in heavy metal metabolism and organic compound inactivation. Consequently, *P. lasiusi* represents a novel species with an intriguing evolutionary history, manifesting in distinctive genomic, metabolomic, and physiological characteristics. This species may have potential applications in the bioaugmentation of contaminated soils.

## 1. Introduction

Ants represent one of the most successful and pervasive taxonomic groups on Earth. The evolutionary history of ants can be traced back to the early Cretaceous period, approximately 140–130 million years ago, when the first flowering plants emerged on Earth. At the present time, there are more than 13,000 species of ants on Earth, which inhabit nearly every region of the planet, with the exception of Antarctica and a few islands. Ants play a pivotal role in the biosphere, influencing the distribution of plants and other animals, as well as soil processes [1].

The black garden ant (*Lasius niger*) plays a pivotal role in ecological processes due to its adaptability and ability to thrive in diverse habitats. Their capacity to establish colonies in soil, beneath stones, or within decomposing wood enables them to be found in close proximity to human habitation. *L. niger* plays a role in ecosystem functioning in a number of ways. First and foremost, these ants serve as vital seed dispersers, facilitating the dissemination and germination of plant species. Secondly, they facilitate soil aeration, thereby enhancing nutrient availability and soil structure. Thirdly, *L. niger* engages in mutualistic relationships with aphids by feeding on the honeydew produced by these insects. This symbiotic relationship benefits both species and helps maintain a balance within the ecosystem. Finally, *L. niger* occupies a pivotal position in the food web. They serve as a vital food source for various invertebrates and birds, contributing to the overall biodiversity and functioning of the ecosystem [2].

The current state of knowledge regarding the interaction of *L. niger* with microorganisms is limited. For example, *Staphylococcus xylosus*, which inhabits the gut and honeydew of *Aphis fabae*, has been demonstrated to release compounds that strongly attract *L. niger*, which in turn provides protection for the aphids from predators [3,4,5]. In another study, members of the family Bacillaceae and the genus *Streptomyces* were found to inhabit *L. niger* anthills [6]. The compounds they produce, including actinomycins, are likely to be beneficial for ants in protecting the nest from potentially pathogenic bacteria and fungi [6]. In a separate study conducted on the closely related ant species *L. fuliginosus*, microbial communities associated with larvae and adults were analyzed using 16S rRNA sequencing [7]. The results revealed that the microbial communities encompass representatives from 20 phyla, with Proteobacteria being the most abundant. Moreover, the bacterial communities associated with the nest carton included bacteria typically found in soil and dead wood, as well as two well-characterized endosymbiotic bacteria, *Rickettsia* and *Wolbachia* [7].

*Arthrobacter* is a genus of obligate aerobic, non-spore-forming, Gram-positive bacteria that belongs to the Micrococcaceae family, which is within the Actinobacteria phylum. At present, approximately 118 fully described species are included in the *Arthrobacter* sensu lato classification, of which 75 are members of the genus Arthrobacter. This group of bacteria is highly diverse in terms of both its geographical distribution on Earth and the ecological niches it occupies. *Arthrobacter* species are commonly found in soil and in association with plant rhizospheres [8,9,10]. An intriguing characteristic of *Arthrobacter sensu lato* representatives is their capacity to biodegrade organic pollutants, which suggests a potential practical application for them. Additionally, a significant number of species were initially isolated from highly contaminated soils and wastewater (e.g., *G. soli*, *Pseudarthrobacter defluvii*, *A. nitrophenolicus*, *Paeniglutamicibacter quisquiliarum*) [11,12,13]. The data indicate that Arthrobacter species may have potential applications in biotechnology, particularly in the bioaugmentation of contaminated soils and the development of plant growth-stimulating components of the rhizosphere.

The current systematics of the genus *Arthrobacter* have been shaped by phylogenetic analyses, which have led to the reclassification of certain species in 2016 and the proposal of novel genera such as *Paenarthrobacter*, *Pseudarthrobacter*, *Glutamicibacter*, *Paeniglutamicibacter*, *Pseudoglutamicibacter*, and *Falsarthrobacter*. Furthermore, the genus has been divided into discrete groups based on robust phylogenetic clusters, high 16S rRNA gene sequence similarities, and group-specific chemotaxonomic traits [14]. Subsequently, numerous additional species within the genus *Arthrobacter* and its derived genera (collectively designated as *Arthrobacter sensu lato*) have been identified through the application of contemporary molecular biological methodologies.

In this study, we identify a novel species, *Paenarthrobacter lasiusi*, isolated from the soil of an *L. niger* anthill. The characterization of *P. lasiusi* was conducted using a combination of microbiological, physiological, biochemical, and molecular methods.

## 2. Results

### 2.1. Genome-Based Identification of the S21 Strain

The genome assembly of S21 was conducted using the SPAdes assembler, resulting in 145 contigs with an N50 of 291,276 bp and a total length of 4,662,005 bp. The maximum contig length was 713,417 base pairs (bp), and the guanine plus cytosine (G + C) content was 62.8873%. The quality of the genome was evaluated using MiGA [15], with a completeness score of 97.2% and a contamination score of 1.9%.

**Figure 1:**
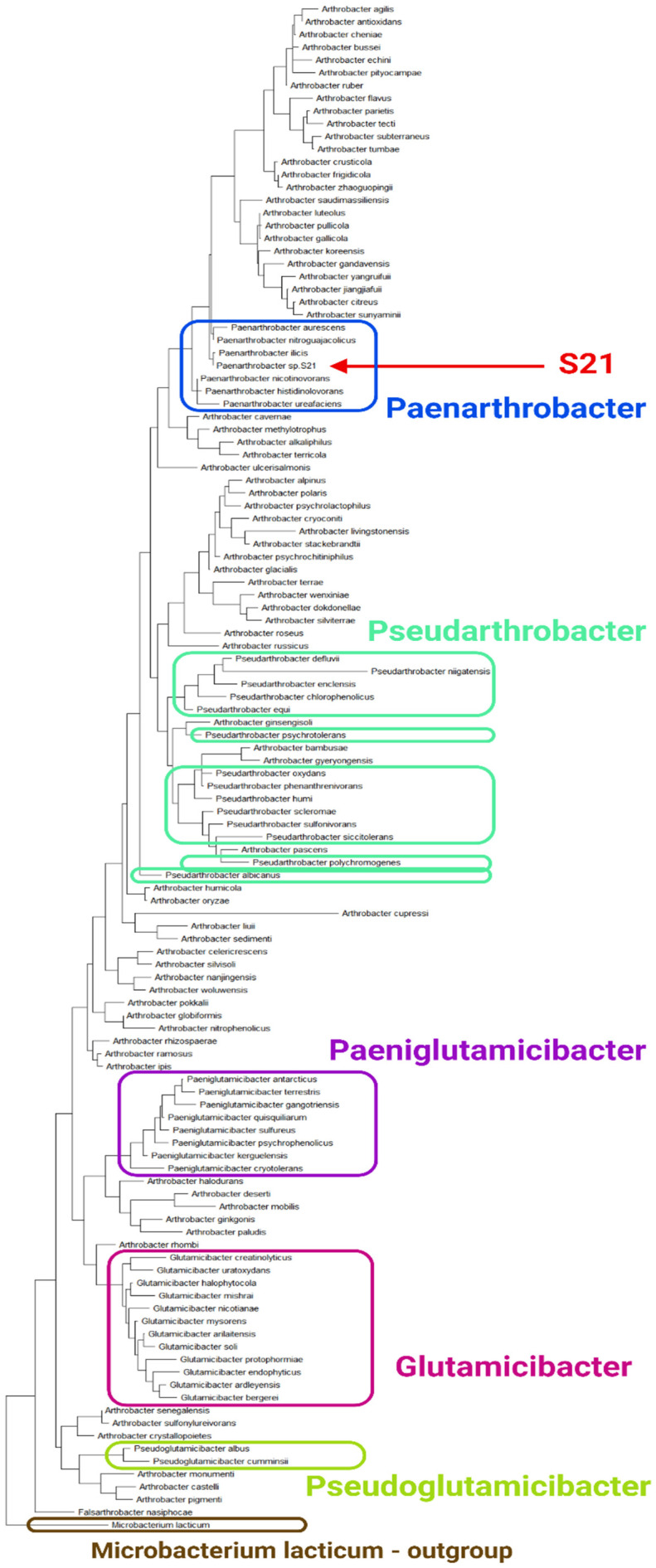
The phylogenetic tree of bacteria from the genera *Arthrobacter*, *Paenarthrobacter*, *Pseudarthrobacter*, *Glutamicibacter*, *Paeniglutamicibacter*, *Pseudoglutamicibacter*, and *Falsarthrobacter* based on the 16S rRNA gene sequences. The outgroup is *Microbacterium lacticum*. Additionally, the names of the genera are indicated. The tree was created using MEGA-X.

A preliminary taxonomic analysis conducted using MiGA identified the strain as belonging to Actinomycetota, Actinomycetes, Micrococcales, and Micrococcaceae. The most closely related strains were identified as belonging to the genera *Arthrobacter* and *Paenarthrobacter*, with an average nucleotide identity (ANI) of approximately 84.5% among the most closely related representatives. To conduct a more precise phylogenetic analysis and taxonomic classification of strain S21, the 16S rRNA gene sequence was utilized. The 16S rRNA gene sequences of bacteria belonging to the genus Arthrobacter and related genera were collated. Additionally, the following bacterial genera were considered: *Paenarthrobacter, Pseudarthrobacter, Glutamicibacter, Paeniglutamicibacter, Pseudoglutamicibacter*, and *Falsarthrobacter*. A total of 119 sequences were utilized, with one sequence extracted from each described species. The majority of the sequences were obtained from the NCBI Nucleotide database, and their accession numbers are provided in Table S1. In the case of several species as well as for S21, the 16S rRNA sequences were identified through the use of sequenced genomes. These predicted sequences are presented in Table S2. Subsequently, the 16S rRNA gene sequences were aligned using the ClustalW 2.0 program [16] to identify conserved regions and sequence variations. The resulting alignment was then utilized to construct a phylogenetic tree, employing the maximum likelihood algorithm within MEGA-X software (version 11.0.13) [17]. The outgroup employed was *Microbacterium lacticum* (Figure 1). The results of this analysis indicate that *Paenarthrobacter, Glutamicibacter, Paeniglutamicibacter*, and *Pseudoglutamicibacter* may be considered monophyletic genera. In contrast to *Falsarthrobacter*, which is a monophyletic genus comprising a single member, *Pseudarthrobacter* is a polyphyletic group, while *Arthrobacter* is a paraphyletic taxon. The most significant finding of this analysis is that *Paenarthrobacter* is not only a valid monophyletic genus, but also that S21 is clustered together with the other members of this genus, and therefore belongs to it.

**Figure 2:**
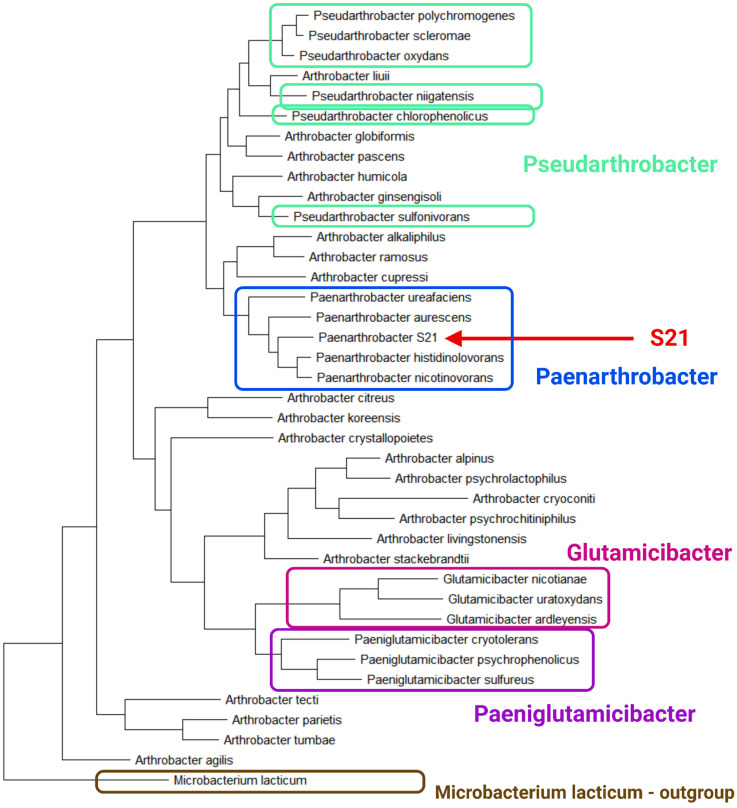
The phylogenetic tree of bacteria from the genera *Arthrobacter*, *Paenarthrobacter*, *Pseudarthrobacter*, *Glutamicibacter*, and *Paeniglutamicibacter* based on concatenated housekeeping genes (*atpD*, *fusA*, *recA*, *rpoB*, *secY*, *tuf*). The outgroup is *Microbacterium lacticum*. Additionally, the names of the genera are indicated. The tree was created using MEGA-X.

In some cases, the resolution of 16S rRNA sequences is insufficient for unambiguous species classification (e.g., in the genus *Bacillus* [18]). It is widely acknowledged that housekeeping genes, due to their less conservative nature in bacterial phylogenetics, provide superior resolution compared to the 16S rRNA gene. Therefore, in the case of Arthrobacter and related genera, six housekeeping genes (*atpD*, *fusA*, *recA*, *rpoB*, *secY*, and *tuf*) were selected to corroborate the data obtained through 16S rRNA analysis [19]. Orthologues of these six genes were identified in the genome of S21 and were used to construct the phylogenetic tree using ClustalW2.0 and MEGA-X software (Figure 2). *M. lacticum* was again used as an outgroup.

The conclusions derived from this analysis are similar to those previously drawn from the analysis of the 16S rDNA gene. The monophyletic groups *Paenarthrobacter, Glutamicibacter,* and *Paeniglutamicibacter* are confirmed, and the polyphyletic *Pseudarthrobacter*, the paraphyletic *Arthrobacter*, and the unrepresented *Falsarthrobacter* and *Pseudoglutamicibacter* are also supported by the analysis. The strain S21 is once again shown to be clustered within the genus *Paenarthrobacter*, thus confirming its genetic identity.

**Figure 3:**
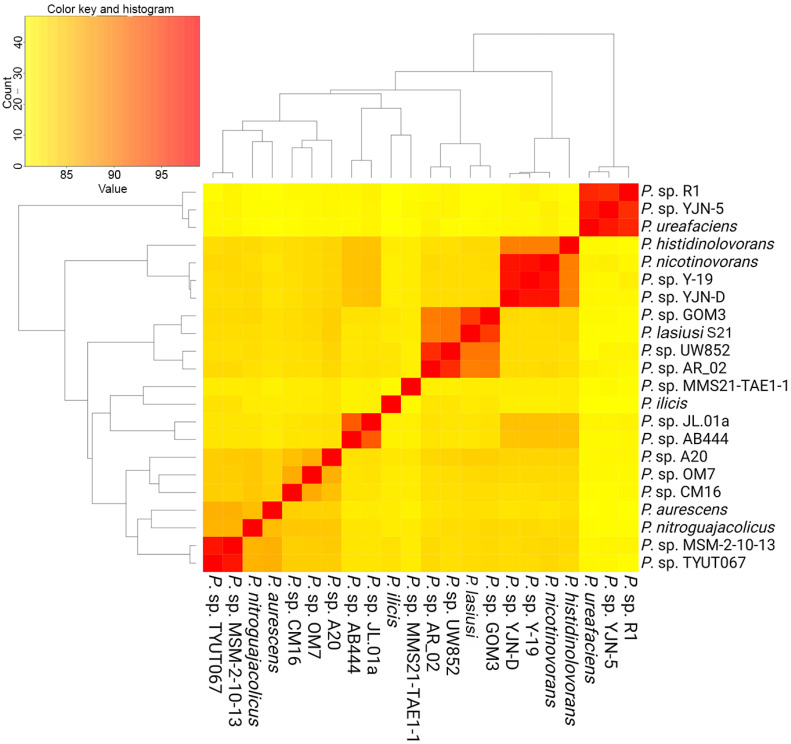
The heatmap of the ANI values between bacterial genomes of the genus *Paenarthrobacter*, as deposited at NCBI. The heatmap is based on the ANI matrix, which was calculated using the FastANI algorithm and is presented in Table S4.

The genus *Paenarthrobacter* comprises six fully described species (*P. aurescens*, *P. ilicis*, *P. histidinolovorans*, *P. nicotinovorans*, *P. nitroguajacolicus*, and *P. ureafaciens*) and 15 strains that have not been described as separate species (*Paenarthrobacter* sp. A20, *Paenarthrobacter* sp. AB444, and others). The following strains have been identified: *Paenarthrobacter* sp. AR 02, *Paenarthrobacter* sp. CM16, *Paenarthrobacter* sp. GOM3, *Paenarthrobacter* sp. JL.01a, *Paenarthrobacter* sp. MMS21-TAE1-1, *Paenarthrobacter* sp. MSM-2-10-13, *Paenarthrobacter* sp. OM7, *Enarthrobacter* sp. R1, *Paenarthrobacter* sp. TYUT067, *Paenarthrobacter* sp. UW852, *Paenarthrobacter* sp. Y-19, *Paenarthrobacter* sp. YJN-5, and *Paenarthrobacter* sp. YJN-D. Their genomes are available in the NCBI database (a comprehensive list of genomes utilized can be found in Table S3). In order to elucidate the phylogenetic relationships between all of the aforementioned species, an ANI matrix was constructed (Figure 3). The ANI method is the most objective method for studying the phylogenetic relationships of bacteria with their complex patterns of evolution and for defining taxon boundaries, particularly at the lower taxonomic levels. The method functions by comparing the nucleotide sequences of two genomes, dividing them into fixed-length fragments (e.g., 1020 bp each), and identifying homologous regions between the genomes through the use of tools such as BLASTn [20]. Subsequently, the ANI value is calculated based on the percentage of identical nucleotides between the aforementioned homologous fragments. A strain that is identical to itself will have an ANI value of 100%. Strains with an ANI value below 95% can be considered to belong to different species. This boundary is not always clearly defined, and researchers may vary it for reasons of expediency. However, it is notable that when ANI drops below 95%, which corresponds to the formal species threshold, there is a significant change in the phenotypic characteristics of bacteria. This suggests that this boundary is objective in a large number of cases [21]. The ANI matrix was constructed using the FastANI algorithm, which enables the rapid execution of a substantial number of calculations and is integrated into the PanExplorer server [22]. The strains of *Paenarthrobacter* sp. PH39-S1 and *Paenarthrobacter* sp. DKR-5 were excluded from the analysis at this preliminary stage, as they exhibited very low ANI values with all other strains of the genus.

The results of the analysis indicate that the ANI value for strain S21 and any of the six *Paenarthrobacter* species is less than 95% and, on average, approximately 85%. This provides substantial evidence that the strain S21 does not belong to any of the currently described *Paenarthrobacter* species. It is therefore designated as *Paenarthrobacter lasiusi* in the following text. Concurrently, the most closely related strain to *P. lasiusi* is *Paenarthrobacter* sp. GOM3 (ANI = 95.6712%), which was recovered from the bottom of the Gulf of Mexico [23]. In accordance with the established criteria, *P. lasiusi* and *Paenarthrobacter* sp. GOM3 are either considered to belong to the same species or are identified as two closely related species. They are also strongly associated with another potential undescribed species (ANI~90%), which consists of strains *Paenarthrobacter* sp. AR02 and *Paenarthrobacter* sp. UW852, the ANI between which also slightly exceeds 95%.

In addition to constructing the ANI Heatmap, we employed digital DNA–DNA hybridization (dDDH) as an additional criterion for comparing the genomes of bacteria belonging to the genus *Paenarthrobacter*. The dDDH algorithm functions by aligning the genome sequences of two organisms and identifying regions of similarity between them. Subsequently, a similarity score is calculated based on factors such as sequence identity and alignment length. By comparing these similarity scores, the dDDH algorithm provides an estimate of the genetic relatedness between the two genomes. Together with ANI, this approach allows for rapid and efficient comparison of large genomic datasets, thus aiding in the taxonomic classification of microorganisms [24]. The dDDH was performed on the GGDC website [25]. The dDDH algorithm employs three distinct formulas, each of which yields slightly different analytical outcomes. In the case of draft genomes, it is recommended that the results from Formula 2 be given priority, as this is the only one that is independent of genome length, in accordance with the guidelines on the GGDC. A value of dDDH = 70% is considered a threshold below which two bacteria can be considered distinct species, analogous to the 95% value selected as the threshold for the ANI method [24]. As shown in Table 1, the strain most closely related to *P. lasiusi* is *Paenarthrobacter* sp. GOM3. Concurrently, while the values yielded by formulas 1 and 3 (dDDH = 80.7% and 80%, respectively) indicate that their relatedness is at the species boundary, the result of formula 2 (dDDH = 63.3%) suggests that these two species can be considered distinct.

In conclusion, based on the formal criteria of ANI and dDDH, it can be stated that *P. lasiusi* is a novel bacterial species. We proposed the name of this species in consideration of its potential symbiotic relationship with ants. The following sections will present a comprehensive physiological and multi-omics analysis of this bacteria, employing genomics and lipidomics approaches.

### 2.2. Annotation of the P. lasiusi S21 Genome

**Figure 4:**
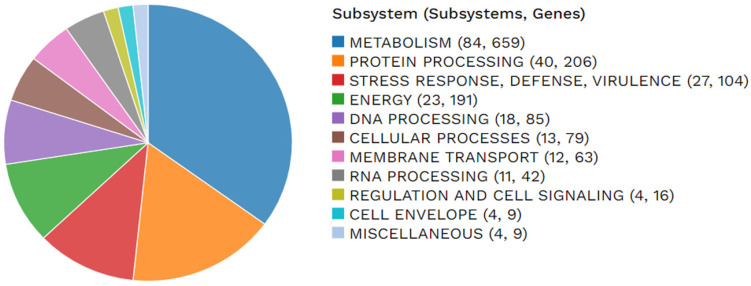
Overall statistics for genes encoding *P. lasiusi* S21 subsystems.

In order to perform genome annotation of the *P. lasiusi* S21 genome, three tools were employed. The following software was utilized for genome annotation: DFAST [26,27], Prokka [28], and RASTtk [29]. The DFAST tool was used on the DFAST server, Prokka on the Proksee server [30], and RASTtk on the BV-BCR server [31]. DFAST identified 4376 coding DNA sequences (CDSs), Prokka identified 4386 CDSs, and RASTtk identified 4531 CDSs. All three tools also identified 54 tRNA genes and 3 rRNA genes. A total of 1747 hypothetical proteins and 2784 proteins with functional assignments were identified among the CDSs discovered by RASTtk. Among the latter group, 996 proteins were assigned Enzyme Commission numbers [32], and 874 proteins were assigned Gene Ontology identifiers. Additionally, 795 proteins were linked to the KEGG pathways [33]. Moreover, 3356 proteins were identified as belonging to genus-specific protein families, and 3898 proteins were classified as cross-genus protein families [34]. Finally, subsystems, defined as sets of proteins that collectively perform a specific function, were analyzed [35]. The results are presented in Figure 4. Additionally, a comprehensive genome analysis was conducted on the determinants of pathogenicity using the BV-BCR server. The results of the DFAST annotation are summarized in Table S5 and Supplementary File S1.

### 2.3. Genes for Mineral Uptake and Heavy Metal Metabolism

As a soil bacterium, *P. lasiusi* S21 should possess a set of genes that allow it to obtain macro- and microelements from soil for its vital activity. Therefore, we searched for genes encoding proteins that ensure the uptake of nitrogen, phosphorus, sulfur, and metals (including heavy metals). The results of the search are presented in Table 2.

Thus, *P. lasiusi* S21, being a typical soil bacterium, can assimilate nitrogen from nitrate, sulfur from sulfate and phosphorus in the form of phosphate. It can also obtain sodium, potassium, and magnesium ions from the external environment. Meanwhile, we found no genes related to molecular nitrogen fixation, including molybdenum-, vanadium-, or ferrum-containing nitrogenases. The presence of heavy metal exporter genes, especially cobalt and nickel, in the genome of *P. lasiusi* S21 indicates its ability to withstand heavy metal pollution conditions.

### 2.4. Genes Involved in Organic Compound Degradation

As we have already noted, many representatives of *Arthrobacter* sensu lato, including a large number of bacteria from the genus *Paenarthrobacter*, are able to degrade organic pollutants, which makes them promising for use in bioaugmentation. Among them, Paenarthrobacter sp. GOM3, genetically closest to *P. lasiusi* S21, is able to degrade monoaromatic compounds such as benzoic acid (via enzymes encoded by the *benABCD* and *catABC* operons), catechol (*catABC* operon), genentisate (via enzymes encoded by the *nagLX*, FAH, and *gtdA* genes), and protocatechuate (via *pcaGHBLDJ* and *fadA* genes) [23]. We used genome annotation results to search for homologous genes in *P. lasiusi* S21. The results of the search are presented in Table 3.

**Figure 5:**
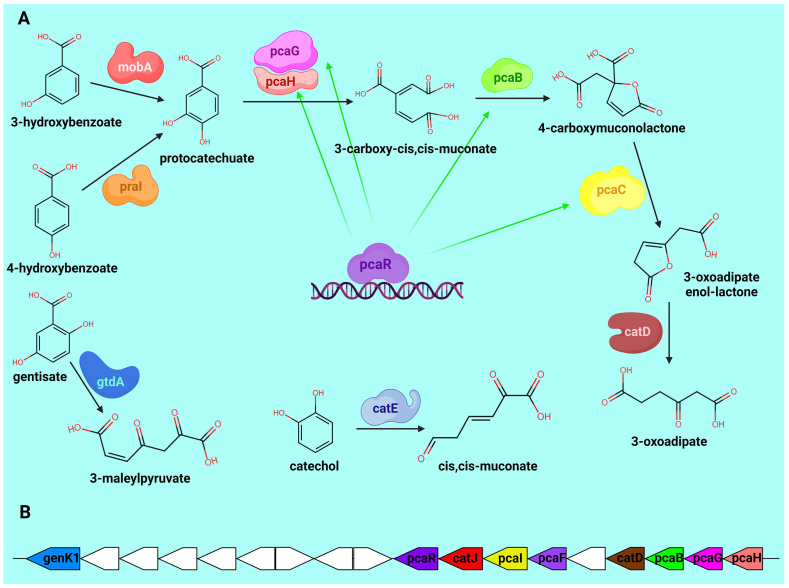
Enzyme genes and the degradation reactions of aromatic compounds catalyzed by them. (**A**) Biochemical reactions for the degradation of aromatic compounds that can be carried out by several enzymes encoded by the *P. lasiusi* S21 genome. (**B**) Cluster of genes involved in the degradation of organic compounds found in contig 4.

Our results show that the genome of *P. lasiusi* S21 contains quite a large number of genes that ensure degradation of organic pollutants. The set of reactions that can be performed by these enzymes is presented in Figure 5A. Moreover, most of the genes found are grouped into a single operon located on contig 4. This is shown in Figure 5B. However, we did not find any of the *benA*, *benB*, *benC*, or *benD* genes that are present in the *Paenarthrobacter* sp. GOM3 genome—only a few benzoate transporters. Thus, bioinformatics analysis predicts a high potential of *P. lasiusi* S21 for bioaugmentation.

### 2.5. Clusters of Secondary Metabolite Biosynthesis Genes in the P. lasiusi S21 Genome

Secondary metabolites synthesized by bacteria determine their ability to assimilate useful substances from the external environment, establish symbiotic relationships with other organisms, and suppress competitors. We used antiSMASH [36] to predict substances that can be synthesized by *P. lasiusi* S21. The results of the search are presented in Table 4 and Supplementary File S2.

If we consider only clusters with similarity equal to or greater than 60% as recommended in the literature [37,38], then the only cluster is reliably predicted for *P. lasiusi* S21. This is the dosferrioxamine E biosynthesis cluster, which is a siderophore characteristic of the genus *Streptomyces*, which, like the genus *Arthrobacter* sensu lato, belongs to the class Actinomycetes [39]. Dosferrioxamine E is essential for the uptake of metals, particularly trivalent elements, such as Fe^3+^, from soil [40,41]. Despite the low 31% similarity, it is possible to consider the other biosynthesis cluster for the biosynthesis of an extremely important compound, namely arthroamide. This is a cyclic depsipeptide discovered in 2015 in a bacterium of the genus *Arthrobacter*. It has the ability to inhibit the growth of other bacteria through the quorum sensing mechanism [42]. It seems plausible to suggest that the biosynthesis of this or a closely related substance by *P. lasiusi* S21 may prove beneficial to ants in suppressing the growth of pathogenic bacteria in the anthill, in a manner analogous to that observed in other bacteria [6]. Another potentially interesting cluster provides carotenoid synthesis. Given that the colonies of *P. lasiusi* S21 have pale yellow coloration, it can be postulated that this strain is capable of synthesizing carotenoids and/or other compounds that provide similar colors.

### 2.6. Antiviral Defense Systems and the History of P. lasiusi S21 Virus Fighting

One of the most crucial aspects of the existence of the majority of bacteria, if not all, is the ongoing battle against bacteriophages. Bacteriophages are in significantly greater numbers than bacteria and are a principal factor limiting their abundance. Consequently, bacteria have evolved a variety of defense mechanisms, including the well-documented restriction–modification and CRISPR/Cas systems, as well as others that have only recently begun to be studied in detail [43]. Accordingly, we undertook an analysis of the genome of *P. lasiusi* S21 to identify any traces of bacteriophage defense mechanisms. We initially sought to ascertain whether the genome of P. lasiusi harbors prophages, which could serve as both residual evidence of recent infections and potential vectors for horizontal gene transfer. Indeed, VirSorter software (version 2.2.4) [44] was employed to detect the presence of two prophages in the genome, located on the second and eighth contigs and subsequently designated as prophage 2 and prophage 8, respectively. The data for these are presented in Table 5.

As prophages contain a number of “hypothetical proteins,” we employed the DFAST algorithm followed by BLASTP to predict the functions of the proteins encoded in these prophages. The results of the search for prophage 2 are presented in Table S6, and the results for prophage 8 are presented in Table S7. Both prophages contain phage-specific homologues of the following proteins: site-specific tyrosine integrase, HNH endonuclease, excisionase, phage major capsid protein, HK97 gp10 family phage protein, phage portal protein, tail protein, and tail length tape measure protein. Furthermore, prophage 2 has phage-specific homologues of amidase, phage tail tube protein, gp6-like head-tail connector protein, HK97 phage prohead protease, terminase, VRR-Nuc domain protein, hydrolase domain protein, immunity suppressor, and so forth. Furthermore, prophage 8 has phage-specific homologues of endolysin, tail fiber protein, flagellar hook-length control protein, phage tail domain-containing protein, head closure Hc1, phage Gp19/Gp15/Gp42 family protein, capsid cement protein, ClpP-related head maturation protease, terminase large subunit, P27 family phage terminase small subunit, and so forth. In addition to phage-specific genes, we also identified genes of bacterial origin (with homologues in the *Arthrobacter* sensu lato group) on these prophages. In prophage 2, these include, for instance, genes for a single-stranded DNA-binding protein, a DNA alkylation repair protein, an oligoribonuclease, a peptide deformylase, and a GTP 1 type 2 cyclohydrolase, which is likely involved in the stress responses. A greater number of such genes are present on prophage 8. The *pst* operon (*pstABCS* genes), which is required for phosphate production, is an operon encoding components of DNA repair pathways (DNA repair protein RadA, DNA integrity scanning protein DisA, A/G-specific adenine glycosylase, and glycosyl hydrolase) and a set of genes encoding enzymes essential for carbohydrate metabolism, including the C4-dicarboxylate transporter DctA, subunit IIA of the sugar transporter PTS, and the subunits L and DhaK of dihydroxyacetone kinase. Additionally, there is a set of genes involved in stress tolerance and encoding the CsbD family protein and the two glyoxalase genes of the VOC family. The aforementioned genes are present in the genome of *P. lasiusi* S21 in a single copy. Furthermore, orthologues of these genes are present in the genome of the closest strain, *Paenarthrobacter* sp. GOM3. However, they are located outside the only prophage detected in this genome using VirSorter. It seems reasonable to posit that these genes originated in the genome of *P. lasiusi* S21 via transduction from the genome of a phylogenetically close bacterium (belonging to *Arthrobacter* sensu lato). Subsequently, the host orthologues were lost during evolution. To the best of our knowledge, this may represent the first documented instance of horizontal gene transfer by transduction in bacteria, followed by the loss of host genes.

Subsequently, we employed the CRISPRMiner tool [45] to ascertain the presence of the CRISPR/Cas system in the genome of *P. lasiusi* S21. A search employing the PILERCR + HmmScan algorithm returned no results. Conversely, the use of the CRISPR/Cas Finder algorithm yielded more intriguing findings, as presented in Table S8. The search results indicated that neither Cas proteins nor loci with complete CRISPR repeats are currently present in the genome of this bacterium. Nevertheless, six loci were identified in disparate regions of the genome. Five of these loci consisted of a single spacer surrounded by two direct repeats (DR), while the sixth locus, which exhibited the greatest degree of conservation, contained six spacers of varying lengths interspersed by seven DRs. Furthermore, one of the loci demonstrated the presence of three intragenomic targets, which may suggest that the CRISPR/Cas system has undergone a process of “self-annihilation” in the past. This phenomenon may occur in the absence of phage pressure, as the CRISPR/Cas system itself is known to possess certain genotoxic properties [46]. In light of the indirect evidence, it can be posited that the CRISPR/Cas system may have existed in the past, but has since been eliminated as a result of “friendly fire.” Then, in the absence of the CRISPR/Cas system, it is possible that two phages may have invaded the bacterium and subsequently remained in its genome as prophages, as identified by our research team. To the best of our knowledge, CRISPR/Cas systems have not been previously searched for in members of *Arthrobacter* or in close genera.

Next, a search was conducted using the PADLOC tool [47] to identify additional antiphage defense systems. The data are summarized in Table 6, and the full data are provided in Table S9 (tabular form) and Supplementary File S3 (GFF format). In total, 13 defense systems are identified in 12 loci of the genome. Notably, some of the systems are represented in multiple copies.

In the genome of *P. lasiusi* S21, two defense systems were identified as belonging to Wadjet, two to Pycsar, one as a restriction–modification type IV, one as a dXTPase (dGTPase), and one as a phage. The candidate defense systems PDC-S14 and PDC-S02 were classified as DNA modification systems “other,” while four PD-T4-6 systems were identified, one of which contains two proteins with the same name. The abundance of antiphage systems may indicate that *P. lasiusi* S21 is effectively protected from the potential invasion of foreign DNA.

### 2.7. A Search for Horizontal Gene Transfer from the Ant Genome

In a close symbiotic relationship between a eukaryotic host and a prokaryotic symbiont, instances of horizontal gene transfer have been observed. For example, portions of plant genomes can be transferred to their bacterial endophytes (often in a spliced form via mRNA) [48]. Such occurrences are uncommon, rendering them intriguing from an evolutionary, molecular, and symbiogenetic perspective. Accordingly, we conducted a search for evidence of potential gene transfer from *L. niger* to *P. lasiusi* S21. A BLASTN [49] search was conducted using sections of the bacterial genome as a query and a database compiled from the ant genome. Furthermore, a tBLASTn [50] search was conducted using the predicted proteins of *P. lasiusi* S21 and the aforementioned reference database. As a result, both the tblastn and blastn algorithms identified only short, similar sequences (no longer than 25 amino acids or 450 bp in the case of tblastn and blastn, respectively), which are insufficient for substantiating a reasonable assumption of horizontal gene transfer. It is noteworthy that *P. lasiusi* S21 was not identified as an endosymbiont of an ant, but rather in the soil of an anthill. Our findings suggest that the interactions between these organisms may not be symbiotic, or that the *P. lasiusi* S21 strain’s defense mechanisms are effectively inhibiting gene transfer.

### 2.8. A Search for Genes Associated with Human Pathogenicity and Antibiotic Resistance

Given the potential of *P. lasiusi* S21 to be utilized in the remediation of soil organic pollutants and the fact that certain members of the Arthrobacter sensu lato species are known to be pathogenic to humans and farm animals, it is imperative to ascertain the safety profile of this bacterium with respect to human health. Consequently, a comprehensive analysis of the *P. lasiusi* S21 genome was conducted on the BV-BCR server, with a particular focus on the prediction of pathogenicity. Furthermore, the presence of pathogenicity-related genes (VFDB [51], Victors) and antibiotic resistance genes (PATRIC, CARD RGI) was investigated as potential drug targets (Drug Bank [52], TTD [53]) and transporters (TCDB [54]). The number of predicted genes is presented in Table 7. For a detailed description of the results of this analysis, please refer to Table S10.

A search for virulence factors has identified genes encoding the metabolic enzymes isocitrate lyase (*icl*) and dihydroxyacid dehydratase (*ilvD*). Homologues of these enzymes have been linked to the pathogenicity of *Mycobacterium tuberculosis* [55], which also belongs to the class Actinomycetes. Similarly, the situation with antibiotic resistance genes is generally the same: we identified ribosomal proteins, translation and transcription factors, and enzymes involved in amino acid, sugar, and lipid metabolism. The only noteworthy discovery is the *mtrAB* operon, encoding MtrB (a histidine kinase), MtrA (a response regulator), and lipoprotein LpqB (which modulates the activity of Mtr proteins). This system has been described as conferring multidrug resistance to *Mycobacterium* spp. by regulating the expression of multiple genes, such as *dnaA* [56,57]. It is thought to be conserved among actinobacteria [58] and is attributed to provide resistance to alkaline, oxidative, and other stresses [59,60], the regulation of antibiotic production [61], and the coordination of this process with sporulation [62]. It may be the case that representatives of the *Arthrobacter* sensu lato group are able to survive in conditions of extreme organic pollution due to the MtrAB system.

Thus, no specialized genes associated with pathogenicity or antibiotic resistance were identified in the genome of *P. lasiusi* S21. It is important to note that the *Arthrobacter* sensu lato group has been relatively understudied, and cases of infection with these bacteria are rare, with only a few reports available. It is plausible that these are opportunistic bacteria that manifest exclusively in immunocompromised individuals.

### 2.9. Microbiological, Physiological, and Biochemical Characteristics of P. lasiusi S21

#### 2.9.1. Antibiotic Resistance Profile of *P. lasiusi* S21

The presence of antibiotics in the environment has been demonstrated to influence the activity and composition of soil microorganisms, ultimately leading to the emergence of antibiotic resistance. The primary pathways through which antibiotics enter the soil include the use of wastewater on agricultural land and manure fertilization. This hypothesis is supported by growing evidence of the presence of antibiotics used in animal husbandry, plant breeding, medicine, and agriculture in soil samples [63,64,65]. At present, there is a paucity of research examining the ecotoxicity of antibiotics in the environment. Moreover, only a limited number of strains within the Paenarthrobacter genus have been subjected to investigation with regard to their antibiotic properties. Meanwhile, due to the continuous evolution of bacteria, an increasing number of strains are becoming resistant to a wide range of antibiotics [66]. Therefore, we tested the resistance profile of *P. lasiusi* S21 to a series of antibiotics (see Table 8).

Despite the absence of specialized genes responsible for antibiotic resistance, *P. lasiusi* S21 demonstrated resistance to amoxicillin, benzylpenicillin, oxacillin, clindamycin, fusidic acid, and linezolid. It is plausible that the observed resistance is, at least in part, mediated by the MtrAB system. However, it remains a possibility that the genome of *P. lasiusi* S21 may contain novel genes responsible for resistance to penicillins, lincosamides, fusidins, and oxazolidinones.

#### 2.9.2. The Lipidome of *P. lasiusi*

**Figure 6:**
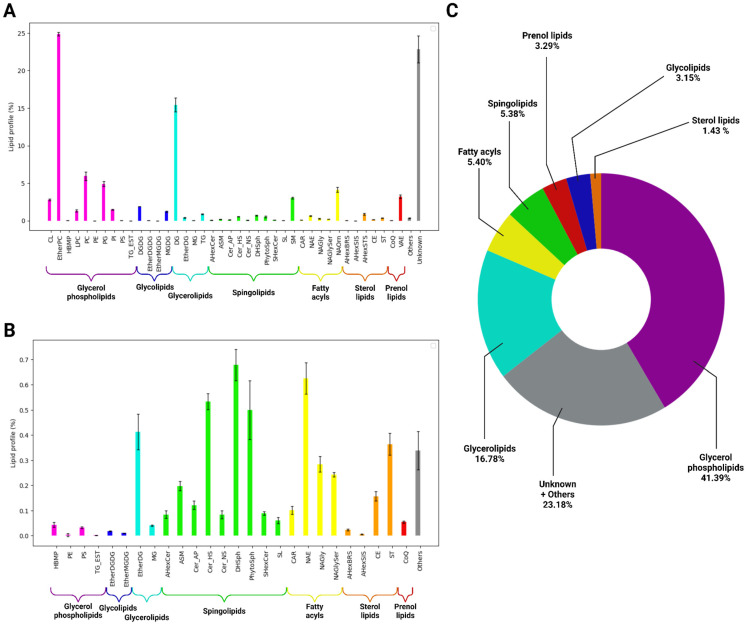
Structure of the lipidome of *P. lasiusi* S21. (**A**) Relative content of 42 abundant lipid subclasses. (**B**) Relative content of 27 minor (<0.7%) lipid subclasses. (**C**) Pie chart of the relative content of the 8 major lipid classes. Abbreviations: CL: Cardiolipin; EtherPC: Ether-linked phosphatidylcholine; HBMP: Hemibismonoacylglycerophosphate; LPC: Lysophophatidylcholine; PC: Phosphatidylcholine; PE: Phosphatidylethanolamine; PG: Phosphatidylglycerol; PI: Phosphatidylinositol; PS: Phosphatidylserine; TG_EST: Triacylglycerol estolides; DGDG: Digalactosyldiacylglycerol; EtherDGDG: Ether-linked digalactosyldiacylglycerol; EtherMGDG: Ether-linked monogalactosyldiacylglycerol; MGDG: Monogalactosyldiacylglycerol; DG: Diacylglycerol; EtherDG: Ether-linked diacylglycerol; MG: Monoacylglycerol; TG: Triacylglycerol; AHexCer: Acylhexosylceramide; ASM: Acylsphingomyelin; Cer_AP: Ceramide alpha-hydroxy fatty acid-phytospingosine; Cer_HS: Ceramide hydroxy fatty acid-sphingosine; Cer_NS: Ceramide non-hydroxyfatty acid-sphingosine; DHSph: Sphinganine; PhytoSph: Phytosphingosine; SHexCer: Sulfatide (from group of Acidic glycosphingolipids); SL: Sulfonolipid (from group of Sphingoid bases); SM: Sphingomyelin; CAR: Acylcarnitine; NAE: N-acyl ethanolamines; NAGly: N-acyl glycine; NAGlySer: N-acyl glycyl serine; NAOrn: N-acyl ornithine; AHexBRS: Acylhexosyl brassicasterol; AHexSIS: Acylhexosyl sitosterol; AHexSTS: Acylhexosyl stigmasterols; CE: Cholesteryl ester; ST: Sterol; CoQ: Coenzyme Q; VAE: Vitamin A fatty acid ester.

The lipidome represents a crucial component of the bacterial cell. Lipids may regulate the essential barrier function of the cellular membrane, thereby mediating resistance to environmental factors, including antibiotics and other organic and inorganic compounds. Consequently, we conducted an analysis of the lipidome of *P. lasiusi* S21 using liquid chromatography coupled with mass spectrometry. A total of 342 lipids and associated compounds were identified, of which 82 were successfully annotated (see Table S11). The relative content of the annotated lipids is presented in Figure 6.

Among all the signals, 22.84% were annotated as “Unknown”. The remainder were dominated by glycerol phospholipids (41.39%), followed by glycerolipids (16.78%), fatty acyls (5.40%), sphingolipids (5.38%), and so forth. Of particular interest were mono- (MGDG) and digalactosyldiacylglycerol (DGDG) and their esters, which were grouped into the glycolipid category (3.15%), and steroid compounds and their derivatives (1.43%), including stigmasterol, brassicasterol, and sitosterol acylhexosyls, which are more commonly found in plants and algae. An abnormally elevated level of diglycerides was also identified, a finding that demonstrates a strong correlation with the presence of glycerol phospholipids and glycolipids, which function as precursors for the aforementioned diglycerides. The level of free fatty acids and their derivatives was found to be comparatively low, a finding that is consistent with the reduced abundance of other classes of lipids that are derived from fatty acids.

#### 2.9.3. The Microbiological and Biochemical Characteristics of *P. lasiusi* S21

*P. lasiusi* S21 is Gram-positive and exhibits growth on nutrient media inherent to soil strains, such as glycerol peptone agar and potato dextrose agar. Optimal conditions for growth are thermostated at 28 °C for 48–72 h. Upon examination through a series of biochemical tests, the strain demonstrated negative results for all except the catalase test, and a response that could be considered controversial with regard to the nitrate reductase activity detection test (Table 9).

Catalase is the most abundant enzyme present in virtually all living organisms exposed to oxygen (such as bacteria, plants, and animals). It catalyzes the decomposition of hydrogen peroxide to water and oxygen [67], thereby protecting organisms from oxidative stress. *P. lasiusi* S21 has been identified as catalase-positive. Additionally, a disk diffusion method was employed to assess the resistance of *P. lasiusi* S21 to hydrogen peroxide. The results demonstrated that the reference strain, *P. ureafaciens* AC-1806, exhibited a complete cessation of growth on media containing 3–12% hydrogen peroxide. In contrast, *P. lasiusi* S21 was observed to form a zone of growth inhibition, with a diameter ranging from 27 to 34 mm. The data indicate that *P. lasiusi* S21 exhibits a more active antioxidant system than a filogenetically similar species.

Nitrogen assimilation is a crucial bacterial process that provides plants and other host organisms with accessible nitrogen forms [68]. The genome of the strain has been found to contain three genes that are related to nitrogen metabolism: nitrogen regulatory protein P-II 1 (GlnB), nitronate monooxygenase, and nitroreductase. The experiment assessing the nitrogen-fixing capacity of *P. lasiusi* S21 demonstrated a low nitrogen-fixing activity of 0.45 (nmol C_2_H_4_/(g × day)). Subsequently, a biochemical test revealed the presence of nitrate reductase activity (Table 9). Nitrate reductase is another crucial enzyme in nitrogen metabolism [69], emphasizing the importance of investigations into the role of soil bacteria in the nitrogen cycle from a practical standpoint.

It is noteworthy that the strain does not produce hemolysin. This finding, in conjunction with the results of the genome analysis, which demonstrated the absence of virulence factors, suggests that this strain is safe for humans.

## 3. Discussion

In the present study, we isolated and comprehensively characterized a novel species of *P. lasiusi* in the genus *Paenarthrobacter*, a bacterium named after the ant *Lasius niger* from whose anthill it was isolated. A distinctive genome feature of *P. lasiusi* S21 is the presence of two prophages that may have horizontally transferred host genes involved in stress responses. The lipidome of *P. lasiusi* S21 contains several lipids that are characteristic of eukaryotes rather than prokaryotes. These include mono- and digalactosyldiacylglycerol, as well as steroid compounds. *P. lasiusi* S21 has been demonstrated to exhibit resistance to penicillins, lincosamides, fusidins, and oxazolidinones. However, no specific genes for resistance to these antibiotics have been identified within the genome. Genomic data and physiological tests indicate that *P. lasiusi* S21 is nonpathogenic to humans. The presence of operons responsible for heavy metal metabolism and organic pollutant inactivation indicates that *P. lasiusi* S21 may be a suitable candidate for soil bioaugmentation.

The genome of *P. lasiusi* S21 has two prophages that carry a set of host stress responsive genes. These genes are absent in other parts of the genome of *P. lasiusi* S21. Moreover, orthologues of these genes are present in the genome of the closest strain, *Paenarthrobacter* sp. GOM3. However, they are not located within the prophage detected in this genome using VirSorter. It seems reasonable to posit that these genes originated in the genome of *P. lasiusi* S21 via transduction from the genome of a phylogenetically close bacterium (belonging to the *Arthrobacter* sensu lato group). Subsequently, the host orthologues were lost during the course of evolution. To the best of our knowledge, this may represent the first documented instance of horizontal gene transfer by transduction in bacteria, followed by the loss of host genes. Furthermore, this may contribute valuable insights into the co-evolution of bacteria and phages. The presence of two prophages with orthologues of host genes in the genome of *P. lasiusi* S21 represents a distinctive genetic feature that differentiates it from *Paenarthrobacter* sp. GOM3. This suggests that these strains have undergone independent evolutionary pathways.

It appears that *P. lasiusi* S21 lost its CRISPR/Cas system following the acquisition of spacers targeting self-genes. However, the genome of *P. lasiusi* S21 contains multiple antiphage systems. The Wadjet system was recently identified through the process of clustering within “defense islands” with other protective systems. The system comprises the *jetA*, *jetB*, and *jetC* genes, which are homologous to the genes encoding condensins *mukF*, *mukE*, and *mukB*, respectively. These are housekeeping genes that are responsible for chromosome segregation during cell division. Additionally, the system includes the *jetD* gene, which is homologous to the gene of topoisomerase IV. All four genes are necessary for the performance of a defensive function, particularly with regard to foreign plasmids in comparison to bacteriophages [70]. The pyrimidine cyclase system of antiphage resistance, PycSA, functions as an abortive infection system. Its activation results in the death of the infected cell, effectively halting the spread of bacteriophages within the population. Two variants of the system have been identified, both of which utilize cyclic pyrimidines as secondary messengers. In one variant, PycC cyclizes CTP, leading to PycTM activation and the disruption of cell membrane function. In the second variant, a PycC protein from a different group specific to UTP forms cUMP, which, upon PycTIR activation, leads to depletion of the NAD+ pool. In both instances, the cell undergoes death [71]. The two systems identified in *P. lasiusi* S21 are of the first type. Another well-characterized system is the dXTPase-based one. It is not an abortive system in the strict sense, as its activation does not result in cell death. However, it functions in a manner that is analogous to an abortive system, deaminating dNTPs and thereby preventing the replication of phages in the affected cell. Additionally, two forms of this system have been identified. In one, dCTP is converted to deoxyuracil nucleotides, while in the other, dGTP is converted to deoxyguanosine [72]. *P. lasiusi* S21 has a dGTPase-based system. Restriction–modification systems have been identified and studied for a considerably longer period of time than the Wadjet, Pycsar, and dXTPase systems described above. Restriction–modification (R-M) systems represent a class of bacterial defense mechanisms comprising restriction enzymes (R) that cleave DNA at specific sequences and methyltransferase enzymes (M) that methylate the same sequences. This dual mechanism serves to protect the host DNA from cleavage. Specifically, type IV R-M systems are methylation-dependent restriction systems that necessitate DNA modification with a methyl group for functionality. The mREase IV protein, which is the sole protein present in type IV R-M systems, is a restriction enzyme that recognizes and cleaves methylated strands of DNA [73]. In contrast, the following systems have been the subject of less rigorous scientific investigation. Of particular interest is PD-T4-6, which was identified within the pangenome of *E. coli* from the prophage region. It comprises a single protein sharing the same designation, which is characterized by a Ser/Thr kinase domain. The mechanism of action of this system remains unknown; however, it has been demonstrated to confer resistance to the phage T4 and related phages T2 and T6 [74]. Our PADLOC results identified five PD-T4-6 systems in the genome of *P. lasiusi* S21. Additionally, we have previously emphasized the high prevalence of these systems in other bacteria, which is a noteworthy observation [38]. Nevertheless, in a study comprising 100 strains of *Pseudomonas aeruginosa*, the PD-T4-6 system was identified in 97 of them. Given that it is monogenic, the authors posited that a considerable proportion of these observations may be false positives [75]. Phage defense candidate systems are distantly related to the previously described antiphage systems. A recent publication reports their presence within the genomes of *P. aeruginosa* [75]. The mechanism of action of such systems has yet to be elucidated. These systems probably actively protect *P. lasiusi* from virus attacks, as well as from horizontal transfer of genetic material from other organisms, including the ant.

An intriguing aspect of the *P. lasiusi* S21 lipidome is the presence of multiple lipids that are typically associated with eukaryotic organisms, rather than prokaryotic ones. These include MGDG, DGDG, and their esters, as well as sterols. MGDG and DGDG are biosynthesized from diacylglycerol through the sequential addition of galactose from UDP-Gal, which is catalyzed by MDGD and DGDG synthases, respectively [76]. These galactolipids are the predominant lipids of the thylakoid membrane in the chloroplasts of plants and algae, and are indispensable for photosynthesis reactions, as they stabilize photosystem II [77,78]. Contemporary data indicate that although cyanobacteria are capable of biosynthesizing MGDG and DGDG, plants and algae did not inherit this pathway directly from cyanobacteria, and their enzymes have diverse origins [79]. It would be of interest to investigate the influence of MGDG and DGDG on the life of other photosynthetic organisms. For example, the *mgdA* gene, which encodes monogalactosyldiacylglycerol synthase, was discovered in the green sulfur bacterium *Chlorobaculum tepidum*. Knockdown of this gene disrupted the assembly of bacteriochlorophyll c, and complete knockout resulted in the inability to isolate a mutant strain, as this gene is essential for bacteria [80]. Information regarding the prevalence of these lipids in other bacterial species is scarce. In 1997, it was documented that the membranes of the bacterium *Bradyrhizobium japonicum*, which forms symbiotic nodules with leguminous plants, contain DGDG. In bacteroids, it is one of the main lipids present in much lower quantities in free-living forms, prompting the authors to consider the potential role of DGDG in the establishment of this bacterium’s symbiosis with plants [81]. Thus, the detection of MGDG, DGDG, and their esters in the membrane of the actinomycete *P. lasiusi* S21 is of interest in itself, as is the fact that the gene encoding MGDG synthase was detected in its genome (LOCUS_06710 in Table S5) initially via blastp, and subsequently corroborated the presence of the corresponding domains for this protein: diacylglycerol glycosyltransferase and glycosyl transferase, MGDG synthase and glycosyltransferase, and MGDG synthase, according to InterPro, Pfam, and PANTHER, respectively. A protein that could serve as a DGDG synthase was not identified, suggesting that this function in the bacterium may be performed by MGDG synthase or an alternative protein distinct from the DGDG synthases included in the analysis.

Sterols are also considered to be primarily lipids of eukaryotes. Cholesterol is a typical component of animal cells, whereas plants possess phytosterols, including β-sitosterol, campesterol, stigmasterol, brassicasterol, and others. Fungi possess ergosterol, fucosterol, and other sterols found in algae. Additionally, various sterols have been described by different protists [82]. It is notable that eukaryotes lacking sterols are considered exceptions, with examples including Tetrahymena infusoria and a number of other protists [83]. In contrast, sterols were previously considered extremely rare in bacteria. For example, the myxobacterium *Plesiocystis pacifica*, *Methylococcus capsulatus*, *Gemmata obscuriglobus*, and *Stigmatella aurantiaca* were only known to produce sterols and possess corresponding genes [82]. Further studies have considerably expanded this list [84]. To date, the steroid biosynthesis pathway has been identified in 14 bacterial phyla, including members of the Actinomycetota ~ Actinobacteria phylum, such as *Streptomyces*, Acidimicrobiaceae, *Nocardia*, *Lentzea,* and *Nonomuraea* genus representatives, as well as Cyanobacteria. The bacterial phyla include Actinobacteria, Chloroflexi, Gemmatimonadetes, Nitrospirae, Rokubacteria, Gammaproteobacteria, Alphaproteobacteria, Bacteroidetes, Planctomycetes, Verrucomicrobia, Acidobacteria, Deltaproteobacteria, and Cyanobacteria. Furthermore, the biosynthesis pathway observed in Myxobacteria (aerobic Deltaproteobacteria) and Dadabacteria is homologous to that observed in eukaryotes (especially plants and algae). It is therefore probable that this pathway was present in the last eukaryotic common ancestor. The current evidence suggests that it was bacteria that “invented” steroids in response to an oxygen burst, while eukaryotic ancestors acquired this pathway via horizontal gene transfer, which enabled them to perform phagocytosis and subsequently obtain mitochondria. This challenges the previously adopted concept [85]. To the best of our knowledge, neither sterols nor hopanoids have yet been identified in archaea [86,87]. Therefore, the occurrence of steroids in bacteria is not as uncommon as previously assumed. Nevertheless, this study represents the first experimental demonstration of the presence of steroids in actinomycetes. Furthermore, no articles have been identified in which compounds traditionally classified as plant steroids, including brassicasterol, sitosterol, and stigmasterol, have been detected in bacterial samples. This may also prove useful in purely applied contexts. For example, brassinosteroids, which are plant hormones with potential applications in agriculture and medicine, are challenging to produce chemically. The use of brassicasterol as a starting compound would greatly simplify the synthesis process; however, it is not sufficiently available and is quite expensive [88]. Another avenue for exploration could be the biotransformation of plant steroids into steroid hormones and their analogues for therapeutic applications, as is currently being undertaken with *Mycolicibacterium* cell factories [89]. Consequently, *P. lasiusi* S21 may be employed as a producer of plant steroids, which may serve as precursors to a range of valuable compounds.

Another potential application of *P. lasiusi* S21 is the bioaugmentation of contaminated soils, as its genome contains multiple operons for heavy metal metabolism and pathways for organic compound destruction. Genome analysis and physiological testing indicate that *P. lasiusi* S21 is safe for humans, making it a valuable candidate for biotechnological applications.

## 4. Conclusions

We identified a novel species, *P. lasiusi*. It was isolated from Lasius niger anthill soil, and its genome contains interesting potential marks of horizontal transfer of bacterial genes by phages, followed by loss of host genes. Another evolutionary unusual feature of *P. lasiusi* S21 genome is the presence of enzymes for the synthesis of lipids that are characteristic of photosynthetic eukaryotes. We hypothesize that this species may have originated as a marine photosynthetic bacterium, and that its transition to a terrestrial habitat (soil) may have resulted in the loss of its photosynthetic machinery. The absence of specialized antibiotic resistance genes could be compensated for by the expression of stress-responsive genes, such as the MtrAB system, which are known to confer antibiotic resistance. However, the possibility cannot be ruled out that the genome of *P. lasiusi* may contain novel antibiotic resistance genes that have yet to be identified. The absence of virulence determinants for humans and the presence of operons responsible for heavy metal and organic pollutant inactivation indicate a high biotechnological potential of *P. lasiusi,* for example, in soil bioaugmentation.

## 5. Materials and Methods

### 5.1. The Isolation of Strain S21 of P. lasiusi from the Anthill of L. niger

Strain S21 was isolated from the central region of the anthill of *L. niger*. All soil samples from the anthills were collected in triplicate in the Kasimov district of the Ryazan region of Russia. The field, which had been removed from agricultural use more than 25 years ago, was located on the high bank of the Unzha River. Previously, the area was utilized primarily for the cultivation of fodder crops, including oats and peas. The structure of the microbial community was determined by inoculating soil samples from the anthills onto a solid agarized glucose-peptone-yeast medium. Prior to further analysis, the soil samples were subjected to a pre-treatment process. A soil suspension with a 1:10 ratio (10 g of soil per 90 mL of water) was treated on a ultrasound machine (USDN-1, “Akadempribor”, Moscow, Russia) for three minutes at a current strength of 0.40 A and a frequency of 15 kHz. The resulting suspensions were diluted 1:100, 1:1000, and 1:10,000, after which 0.1 mL was taken from the middle fraction for seeding to the nutrient medium. The samples were incubated for a period of five days at a temperature of 28 °C.

### 5.2. DNA Isolation, Library Preparation, and Whole-Genome Sequencing

Prior to the isolation of DNA, the bacterial samples were stored at −80 °C. The total DNA was isolated from the bacteria using the DNeasy PowerSoil Pro DNeasy kit (Qiagen, Hilden, Germany) in accordance with the manufacturer’s instructions. The quantity of DNA was determined using a Qubit^®^ 2.0 fluorometer (Thermo Fisher Scientific, Waltham, MA, USA). Approximately 500 ng of genomic DNA was utilized for the preparation of DNA libraries with the KAPA HyperPlus Kit (Roshe Diagnostics, Indianapolis, IN, USA), in accordance with the manufacturer’s instructions. AMPure XP beads were employed for the selection of DNA libraries based on size (400–800 bp). The length distribution of the prepared DNA libraries was verified using gel electrophoresis by a Tape Station analyzer (Agilent Technologies, Santa Clara, CA, USA); then, library concentration was evaluated by a Qubit^®^ 2.0 fluorometer (ThermoFisher Scientific, Waltham, MA, USA). All libraries were sequenced (2 × 76 b.p., 4.3 million pair reads per sample) using a NextSeq 550 Mid output kit on Illumina’s NextSeq platform (Illumina, San Diego, CA, USA), in accordance with the manufacturer’s instructions.

### 5.3. Genome Assembly and Quality Control

The quality of the raw reads was evaluated using FastQC software, version 0.11.8 (https://www.bioinformatics.babraham.ac.uk/projects/fastqc/, accessed 11 March 2024). The raw reads were processed using the trimmomatic 0.39 program with the following parameters: average quality of reads 30 and other default parameters [90]. The genome assembly was conducted using the SPAdes assembler [91] on the Galaxy web service [92] with automatic k-measure size selection, with all other parameters set to their default values. The quality of the draft genome was evaluated using the QUAST 5.2.0 program [93]. The completeness of the genome sequencing was evaluated using the BUSCO 5.4.7 program [94] and CheckM (the latter was employed in the form integrated into the DFAST annotation pipeline) [95]. The assembled contigs were screened for contaminants, including PhiX and human sequences, using the Kraken 2.1.2 tool [96]. Contamination checks were also carried out simultaneously by the BUSCO and CheckM programs while assessing completeness.

### 5.4. Genome Annotation

The assembled genome was annotated using a combination of tools, including DFAST, Prokka on the Proksee server, RASTtk and PATRIC [97] on the BV-BCR server (as parts of the comprehensive genome analysis pipeline), and NCBI prokaryotic genome annotation pipeline (PGAP) [98] as a standalone tool.

The results of the DFAST annotation were primarily utilized for the analyses due to the convenient tabular output format (see Table S5). The results obtained from the aforementioned annotation methods were employed to further validate the results obtained, search for additional genes not identified by DFAST, and search for alternative gene names, among other applications. Additionally, the results of the NCBI PGAP annotation were employed to facilitate the upload of the genome to the NCBI website.

### 5.5. Determination of the Taxonomic Identity of P. lasiusi S21

The MiGA and DFAST web services were employed for the preliminary evaluation of the systematic position of the S21 strain, as they identified the most closely related strains based on the ANI value and facilitated the taxonomic affiliation with genus-level precision. To further elucidate the taxonomic affiliation of the S21 strain, 16S rDNA gene sequences of the *Arthrobacter* sensu lato species were obtained from the NCBI database. The accession numbers of the 16S rDNA genes utilized in the analysis are presented in Table S1. If no such entry was available in the NCBI Nucleotide database, as was the case for *P. lasiusi* S21, the sequences were predicted from the appropriate genomes using DFAST annotation. The predicted sequences are presented in Table S2. The 16S rDNA gene sequence of *M. lacticum* was employed as an outgroup. A multiple alignment was constructed for all sequences using the ClustalW algorithm [99] in the MEGA-X program. A phylogenetic tree was then constructed using the maximum likelihood algorithm, also in MEGA-X software.

An additional phylogenetic tree was constructed using the concatenated sequences of housekeeping proteins. The concatenated protein sequences of *atpD*, *fusA*, *recA*, *rpoB*, *secY*, and *tuf* were utilized for a number of representatives of *Arthrobacter* sensu lato, as described earlier [19]. The NCBI accession numbers of the proteins included in the analysis correspond to those used in this study and are KY827405–KY827812. Homologous proteins of *P. lasiusi* S21, as well as *M. lacticum* (which was taken as an outgroup; here, we used genome assembly ASM671681v1), were predicted using the DFAST annotation and then aligned with proteins from other bacteria using pairwise alignment on the EMBL-EBI server [100], with sequences of strictly defined lengths included in the analysis. The protein sequences were concatenated in Python 3.0 in the specified order, *atpD-fusA-recA-rpoB-secY-tuf*, for each bacterium. Subsequently, a multiple alignment was constructed for these sequences in MEGA-X software using ClustalW. The results of this alignment were employed to construct a phylogenetic tree in MEGA-X using the maximum likelihood algorithm.

The genomes of bacteria belonging to the genus *Paenarthrobacter*, which have been deposited in the NCBI Genome database, were used to calculate the ANI. One genome was selected for analysis from each of the six described species: *P. aurescens*, *P. ilicis*, *P. histidinol*, *P. nitroguajacolicus*, *P. nicotinovorans*, and *P. ureafaciens*. Furthermore, the genomes of *Paenarthrobacter* strains that have not yet been classified, including those designated as *Paenarthrobacter* sp., were also included in the analysis. Subsequently, *Paenarthrobacter* sp. PH39 and *Paenarthrobacter* sp. DKR-5 were excluded from the analysis due to the low ANI values observed with the remaining bacteria. A comprehensive list of the genomes utilized is provided in Table S3. These genomes were uploaded to the PanExplorer server and analyzed using the FastANI algorithm, resulting in a matrix of ANI values between pairs of genomes.

The same genomes were subjected to analysis using the dDDH algorithm. For this purpose, the genomes were uploaded to the GGDC website, with each of the genomes used as a reference for comparison with the genome of *P. lasiusi* S21. In accordance with the recommendations set forth on the website in the FAQs section (“Why do the three distance formulae sometimes yield different results and in which DDH estimate should I trust?”), the dDDH value obtained using formula 2 was used to interpret the results.

### 5.6. Functional Annotation of the P. lasiusi S21 Genome

Genes related to mineral compound uptake (such as nitrogen, sulfur, phosphorus, sodium, potassium, and magnesium), heavy metal tolerance genes (cobalt, nickel, zinc, and cadmium transporters), and aromatic compound biodegradation genes were identified through manual searching of DFAST annotation results and, to a lesser extent, annotation results from other tools. The antiSMASH bacterial version web service was utilized to identify gene clusters responsible for the biosynthesis of secondary metabolites.

Prophages in the genome were identified using the VirSorter program, which is integrated into the Proksee web server. To identify homologous genes in these regions, the BLASTP algorithm was employed on the NCBI website [101]. The CRISPRMiner web service was employed to identify CRISPR systems and their residues, utilising both algorithms. PILERCR + HmmScan and CRISPR/Cas Finder were employed for this purpose. The PADLOC web server was utilized to search for antiphage defense systems.

To identify instances of horizontal gene transfer from *L. niger* to the *P. lasiusi* S21, the *L. niger* genome (https://www.ncbi.nlm.nih.gov/datasets/genome/GCA_001045655.1/, accessed 11 March 2024) was employed as a reference, and both blastn (with the *P. lasiusi* S21 genome as the query) and tblastn (with the *P. lasiusi* S21 proteins as the query) were utilized. The BV-BRC Integrated Genome Analysis pipeline, accessible via the BV-BRC server, was employed for the purpose of predicting antibiotic resistance and pathogenicity phenotypes. This analysis involved the utilization of a multitude of databases, including CARD RGI [102], PATRIC, DrugBank, TTD, TCDB, VFDB, and Victors [103] to perform a comprehensive analysis.

### 5.7. Panoramic Analysis of the Lipidome

#### 5.7.1. Sample Preparation

Prior to analysis, the samples were stored at −80 °C. The samples were thawed on ice. Following the thawing process, the samples were subjected to a 10-s vortexing procedure. An amount of glass beads (0.1–0.2 mm) equivalent to half the volume of the original sample was then added to the samples. Subsequently, 500 µL of the internal standard solution, containing 1-butanol, was added to the samples. The samples were subjected to homogenization using a bead beater homogenizer for a total of three cycles, each lasting 25 s and operating at maximum speed, with a 1–2 min interval between cycles. Subsequently, the samples were subjected to ultrasonication at room temperature for a period of 30 min at the maximum power setting. Subsequently, the samples were transferred to a thermal shaker and incubated for 10 min at 1200 rpm and room temperature. Subsequently, the samples were subjected to centrifugation at 13,000 rpm for 10 min at 15 °C. Following completion of the centrifugation process, 450 µL of the organic layer was collected, lyophilized to dryness, and subsequently reconstituted in 50 µL of mobile phase B (acetonitrile:isopropanol 1:9 + 10 mM ammonium formate). The samples were then subjected to centrifugation at 13,000 rpm for 10 min at 15 °C. From the resulting solution, 40 µL was extracted and transferred to labeled vials with inserts for subsequent analysis.

#### 5.7.2. Sample Analysis

A Sciex 6600QTOF time-of-flight mass spectrometer with a calibrant delivery system (CDS) and an Exion 30AD liquid chromatograph were employed for the analysis. The ion source settings were as follows: TEM = 350 °C; GS1 = 45; GS2 = 45; CUR = 35; IS = 5500. Ion detection was conducted in the positive ionization mode of the sample in the TOFMS mode within the range of 350–1700 *m/z*.

The components of the test sample were subjected to chromatographic separation in RPLC chromatography mode using a Waters ACQUITY C8 2.1 × 100 mm 1.7 μm chromatography column. The mobile phase consisted of a water–acetonitrile (4:6) mixture with 10 mM ammonium formate in phase A and an acetonitrile–isopropanol (1:9) mixture with 10 mM ammonium formate in phase B. The mobile phase consisted of acetonitrile: isopropanol (1:9) with 10 mM ammonium formate, with an injection volume of 10 μL. The gradient program was as follows: 0 min, 10% B; 4 min, 30% B; 5 min, 48% B; 22 min. The mobile phase consisted of 65% B for 24 min, 99% B for 4 min, 99% B for 28.2 min, and 10% B for 10 min, with a flow rate of 0.25 mL/min and a thermostat temperature of 55 °C. Two technical repetitions were conducted for each sample in positive sample ionization mode.

#### 5.7.3. Processing of the Results

The results were processed using SCIEX MasterView and Skyline MSDIAL software (version 24.1). The ions selected for further analysis were subjected to a 20-fold prefiltering process against the blank sample. The MSDIAL software was employed for lipid annotation, utilizing a generated lipid library and the MS-DIAL LipidBlast library (version 68). The relative quantification of annotated lipids was conducted by summing the areas under the peaks corresponding to all ions for a given lipid. The data on the relative content of annotated lipids were visualized using a custom script written in Python 3.5.6.

### 5.8. Biochemical Tests of the P. lasiusi S21

In order to characterize the isolated strain, a series of biochemical tests were performed. The kits utilized for the respective groups of microorganisms facilitate the performance of tests with varying substrates, thereby enabling the assessment of the outcomes of biochemical reactions. The following parameters were subjected to examination: malonate utilization, Voges–Proskauer test, citrate utilization, ONPH, and tests for nitrate reductase, catalase, arginine, sucrose, mannitol, glucose, arabinose, and trehalose metabolism.

The nitrogen-fixing ability of the strain was evaluated by the acetylene reduction technique [104] using a gas chromatograph with a flame ionization detector Crystal-2000 (META-KHROM, Yoshkar-Ola, Russia). Briefly, 1 mL of acetylene was added to a pure overnight culture in LB liquid medium, followed by incubation in a thermostat for 1–2 h. Determination was performed in 5-fold repetition. The nitrogen fixation activity was expressed in nmol C_2_H_4_/(g × day).

The antibiotic resistance of *P. lasiusi* S21 was evaluated through the disk-diffusion method, with the interpretation of the results of growth retardation zone measurements based on the standards set forth by the European Committee on Antimicrobial Susceptibility Testing (EUCAST, version 11.0, valid from 1 January 2021).

The cultures were diluted in sterile distilled water to a turbidity of 0.5 units, in accordance with the McFarland standard, which corresponds to a concentration of 1.5 × 10^8^ Colony Forming Units per milliliter. A 0.1 mL aliquot of the suspension was applied to the surface of Mueller-Hinton agar (Himedia, Maharashtra, India) in order to form a uniform lawn. This was achieved by spreading the aliquot over the surface of the nutrient medium in a Petri dish with a spatula. Subsequently, antimicrobial-treated disks were positioned on the surface after a brief interval for the suspension to be absorbed. Following the application of the disks, the Petri dishes were transferred to a thermostat and incubated at 37 °C for 24 h. Subsequently, the results were recorded by measuring the diameter of the growth retardation zone of the cultures tested in millimeters around the disks. The antibiotics that were tested as antimicrobial agents were as follows: amoxicillin, ampicillin, benzylpenicillin, oxacillin, gentamicin, rifampicin, vancomycin, clindamycin, levomycetin, ciprofloxacin, cefotaxime, ceftazidime, and ceftriaxone. The antimicrobial agents tested included doxycycline, fusidic acid, linezolid, trimethoprim/sulfamethoxazole, ofloxacin, erythromycin, imipenem, and an oxidizing agent that acts as a stressor, namely hydrogen peroxide (35%).

## Figures and Tables

**Table 1:** The results of the comparative genomic analysis of *P. lasiusi* and other genomes within the genus *Paenarthrobacter*, conducted using the dDDH method.

Genome	Formula 1	Formula 2	Formula 3
	dDDH		
*Paenarthrobacter* sp. OM7	49.8	25.9	42.1
*Paenarthrobacter* sp. PH39-S1	13.4	19.9	13.8
*Paenarthrobacter* sp. R1	32.5	22.5	28.9
*Paenarthrobacter* sp. TYU067	46.5	25.3	39.6
*P. ureafaciens*	36	22.6	31.4
*Paenarthrobacter* sp. UW852	71.3	41.8	65.4
*Paenarthrobacter* sp. Y19	53.3	25.1	44
*Paenarthrobacter* sp. YJN-5	31.1	22.6	28
*Paenarthrobacter* sp. YJN-D	54.4	25.1	44.6
*Paenarthrobacter* sp. A20	50.2	26.5	42.7
*Paenarthrobacter* sp. AB444	51.7	24.3	42.4
*Paenarthrobacter* sp. AR02	71.9	40.5	65.3
*P. aurescens*	52.3	24.7	43.1
*Paenarthrobacter* sp. CM16	42.2	25.7	36.9
*Paenarthrobacter* sp. DKR-5	14.3	19.9	14.5
*Paenarthrobacter* sp. GOM3	80.7	63.3	80
*P. histidinolovorans*	52.1	25.3	43.3
*P. ilicis*	48.1	24.3	40.1
*Paenarthrobacter* sp. JL01a	51	24.5	42.1
*Paenarthrobacter* sp. MMS21-TAE1-1	37.9	23.5	33
*Paenarthrobacter* sp. MSM-2-10-13	47.2	25.3	40.1
*P. nicotinovorans*	52.8	25.2	43.7
*P. nitroguajacolicus*	47.5	25.5	40.4

**Table 2:** Genes and their encoded proteins for mineral uptake and heavy metal metabolism in the genome of *P. lasiusi* S21.

Element	The Corresponding Proteins Encoded by the Genome
N	Nitrogen regulatory protein P-II 1 (*glnB*), nitronate monooxygenase, nitroreductase
S	Sulfate ABC transporter permease, sulfate adenylyltransferase (x2, including *cysN*), phosphoadenosine phosphosulfate reductase (*cysH*), sulfite reductase, sulfur carrier protein (*cysO*), sulfurtransferase (x6, including *FdhD*)
P	Inorganic phosphate transporter (x2), phosphate import ATP-binding protein PstB (*pstB*), phosphate transport system permease protein PstA (*pstA*), phosphate transport system permease protein PstC (*pstC*), phosphate-binding protein PstS (*pstS*), phosphate starvation protein PhoH (*phoH*), phosphate transport system regulatory protein PhoU (*phoU*)
Na^+^	Sodium/hydrogen exchanger, sodium–proton antiporter, sodium–solute symporter
K^+^	Potassium transporter, potassium transporter Trk, potassium transporter TrkA (x3), potassium-transporting ATPase ATP-binding subunit (kdpB), potassium-transporting ATPase KdpC subunit (*kdpC*), potassium-transporting ATPase potassium-binding subunit (*kdpA*)
Mg^2+^	Magnesium transporter, magnesium transporter CorA (*corA*)
Ni^2+^/Co^2+^	Ni2+/Co2+ efflux system (*nicT*), dipeptide/oligopeptide/nickel ABC transporter ATP-binding protein (x3), cobalt ABC transporter ATP-binding protein
Zn^2+^	ZIP family zinc transporter

**Table 3:** Genes involved in the destruction of organic compounds in the genome of *P. lasiusi* S21.

Number	Contig	Strand	Positions	Gene Name	Enzyme Name
1	1	+	695,695–696,567	*catE*	glyoxalase
2	2	−	25,986–27,983	*mobA1*	3-hydroxybenzoate 4-monooxygenase
3	2	−	96,371–98,302	*mobA2*	3-hydroxybenzoate 4-monooxygenase
4	4	−	23,800–25,158	*genK1~benK*	gentisate/benzoate MFS transporter
5	4	−	33,919–34,722	*pcaR*	transcriptional regulator
6	4	−	34,830–35,498	*catJ*	3-oxoadipate CoA-transferase subunit B
7	4	−	35,501–36,187	*pcaI*	3-oxoadipate CoA-transferase subunit A
8	4	−	36,190–37,404	*pcaF~fadA1*	acetyl-CoA acyltransferase
9	4	−	37,898–38,701	*catD1*	3-oxoadipate enol-lactonase 2
10	4	−	38,694–40,109	*pcaB*	3-carboxy-cis,cis-muconate cycloisomerase
11	4	−	40,106–40,690	*pcaG*	protocatechuate 3,4-dioxygenase subunit alpha
12	4	−	40,695–41,564	*pcaH*	protocatechuate 3,4-dioxygenase subunit beta
13	4	+	113,467–113,901	*catD2*	oxidoreductase
14	8	−	48,909–50,207	*genK2~benK*	gentisate/benzoate MFS transporter
15	8	+	51,267–52,403	*sdgD~gtdA*	gentisate 1,2-dioxygenase
16	9	−	35,024–35,725	*pcaJ*	succinyl-CoA--3-ketoacid-CoA transferase
17	10	+	81,336–82,553		benzoate transporter
18	20	−	29,256–29,591	*pcaC1*	4-carboxymuconolactone decarboxylase
19	20	−	34,939–35,280	*pcaC2*	4-carboxymuconolactone decarboxylase
20	21	−	6250–6987	*nagL*	maleylpyruvate isomerase
21	31	−	225–1412	*praI~pobA*	4-hydroxybenzoate 3-monooxygenase
22	31	+	2506–3723	*genK3*	MFS transporter

**Table 4:** Clusters of secondary metabolite biosynthesis genes found in the genome of *P. lasiusi* S21.

Region	Coordinates (bp)	Type	Most Similar Known Cluster (Species Name)	Similarity
Region 8.2	105,348–117,189	NRPS-independent siderophore	Desferrioxamine E (*Streptomyces* sp. ID38640)	100%
Region 21.1	3250–37,188	NAPAA	Stenothricin (*Streptomyces filamentosus* NRRL 15998)	31%
Region 8.1	52,894–73,799	Terpene	Carotenoid (*Brevibacterium linens*)	28%
Region 13.1	9428–34,719	Betalactone	Microansamycin (*Micromonospora* sp. HK160111)	7%

**Table 5:** Prophages detected in the genome of *P. lasiusi* S21.

Name of Prophage	Group	Contig	VirSorter Score	Coordinates (bp)	Length, bp	Gene Number
1 (“prophage 2”)	dsDNA	2	0.933	498,442–544,606	46,165	59
2 (“prophage 8”)	dsDNA	8	0.913	162,397–232,028	69,632	92

**Table 6:** Antiviral defense systems found in the genome of *P. lasiusi* S21.

Number	Contig	Strand	Positions	Name of the System	Effector Proteins
1	14	+	62,088–67,931	Wadjet	JetA, JetB, JetC
2	20	+	9754–16,587	Wadjet (Type I)	JetA1, JetB1, JetC1, JetD1
3	6	−	231,374–232,872	Pycsar (effector)	PycTM, PycC
4	22	−	52,004–53,223	Pycsar (effector)	PycTM, PycC
5	3	−	20,807–22,123	dXTPase	dGTPase
6	3	−	389,783–392,974	R-M (type IV)	mREase IV
7	3	−	384,390–385,346	PDC-S14	PDC-S14
8	2	+	430,173–431,261	PDC-S02	PDC-S02
9	2	+/−	153,126–160,035	DMS (other)	SngC, mad3
10	4	−	220,459–224,232	PD-T4-6	PD-T4-6 (x2)
11	5	+	143,525–145,510	PD-T4-6	PD-T4-6
12	12	−	87,289–88,824	PD-T4-6	PD-T4-6
13	29	+	6219–7079	PD-T4-6	PD-T4-6

**Table 7:** A summary of the genes predicted to have medical relevance in *P. lasiusi* S21.

Category	Virulence Factor			Antibiotic Resistance		Transporter	Drug Target	
Algorithm	VFDB	Victors	PATRIC_VF	PATRIC	CARD	TCDB	DrugBank	TTD
Number of genes found	1	1	2	35	1	5	11	2

**Table 8:** Antibiotic resistance profile of *P. lasiusi* S21.

Antibiotic Group	Antibiotic	Diameter of the Growth Inhibition Zone	Sensitivity (S)/Resistance (R)
Penicillins	Ampicillin	<19	R
	Benzylpenicillin	0	R
	Oxacilin	0	R
	Amoxiclav	0	R
Cephalosporins	Ceftriaxone	<32	S
	Cefotaxime	<30	S
	Ceftazidime	<23	S
Carbapenems	Imipenem	<24	S
Aminoglycosides	Gentamicin	<14	S
Fluoroquinolones	Ciprofloxacin	<23	S
	Ofloxacin	<23	S
Tetracyclines	Doxycycline	<25	S
Macrolides	Imipenem	<24	S
Diaminopyrimidines/sulfonamides	Trimethoprim/ sulfomethoxazole	<22	S
Amphenicols	Levomecitin	<32	S
Lincosamides	Clindamycin	0	R
Fusidins	Fusidic acid	0	R
Oxazolidinones	Linezolid	0	R
Rifamycins	Rifampicin	<32	S
Glycopeptides	Vancomycin	<16	S

**Table 9:** Biochemical test results for *P. lasiusi* S21.

Name of Test	Result
Ability to utilize citrate as a sole carbon source	−
Ability to utilize malonate as a sole carbon source	−
Acetoin detection (Voges–Proskauer test)	−
Detection of I-galctosidase activity (ONPH test)	−
Detection of nitrate reductase activity	−/+
Detection of catalase activity	+
Arginine decarboxylase detection	−
Sucrose utilization test	−
Mannitol utilization test	−
Glucose utilization test	−
Arabinose utilization test	−
Trehalose utilization test	−
Desoxyribonuclease detection test	−
Ribonuclease activity detection test	−
Hemolysin detection test	−

## Data Availability

All data generated during and/or analyzed during the current study are available from the corresponding author on reasonable request.

## Supplementary Materials

The following supporting information can be downloaded at: https://www.mdpi.com/article/10.3390/ijms26010067/s1.
